# Supplementary figures and images for: NAT2 global landscape: Genetic diversity and acetylation statuses from a systematic review
Source: PLoS One. 2023 Apr 6;18(4):e0283726. doi: 10.1371/journal.pone.0283726 (PMC10079069; doi:10.1371/journal.pone.0283726)

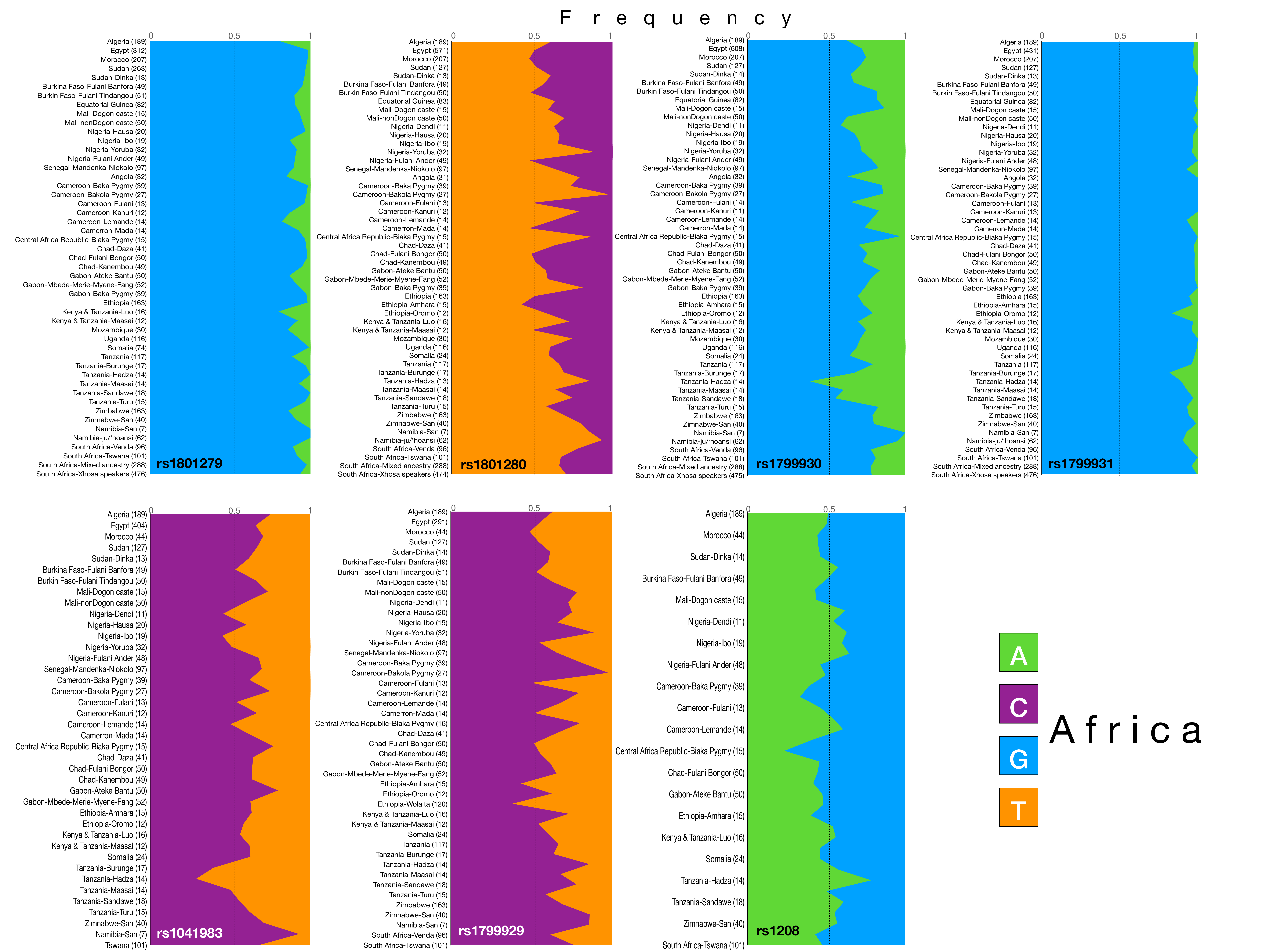

Supplement: S1 Fig — Note: A, Adenine; C, Cytosine, G, Guanine; T, Thymine. (TIFF) [file pone.0283726.s001.tiff]

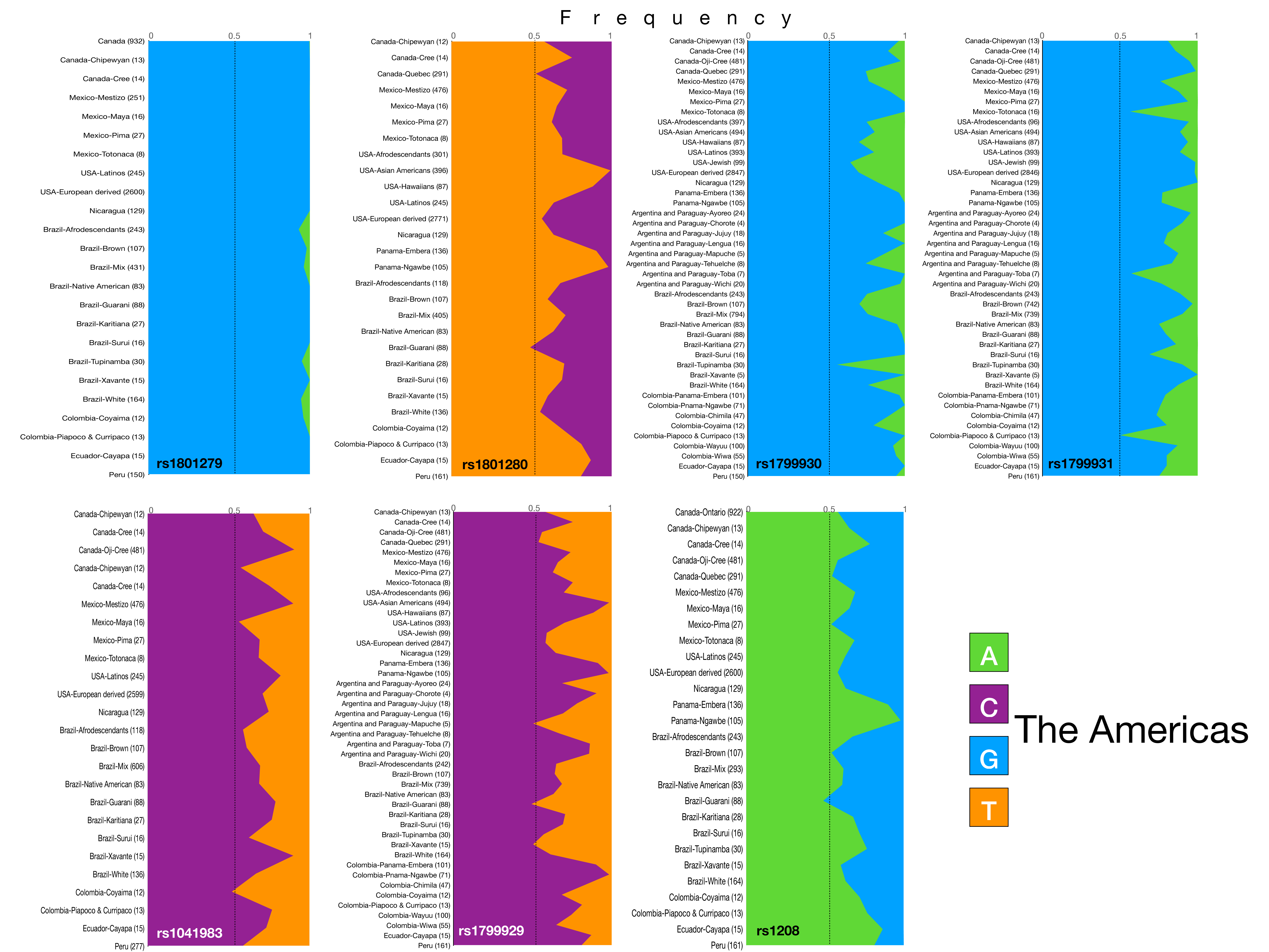

Supplement: S2 Fig — Note: A, Adenine; C, Cytosine, G, Guanine; T, Thymine. (TIFF) [file pone.0283726.s002.tiff]

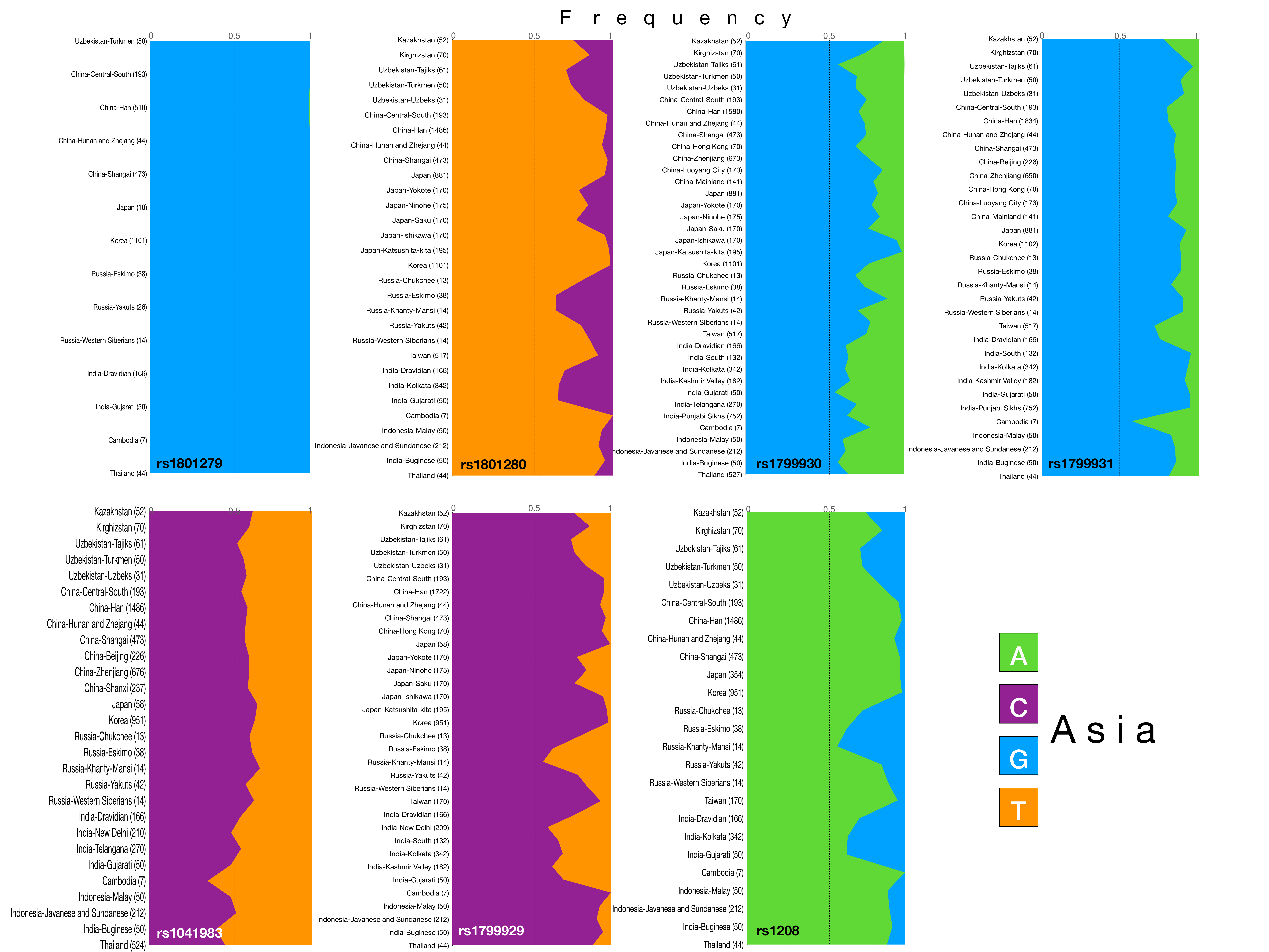

Supplement: S3 Fig — Note: A, Adenine; C, Cytosine, G, Guanine; T, Thymine. (TIFF) [file pone.0283726.s003.tiff]

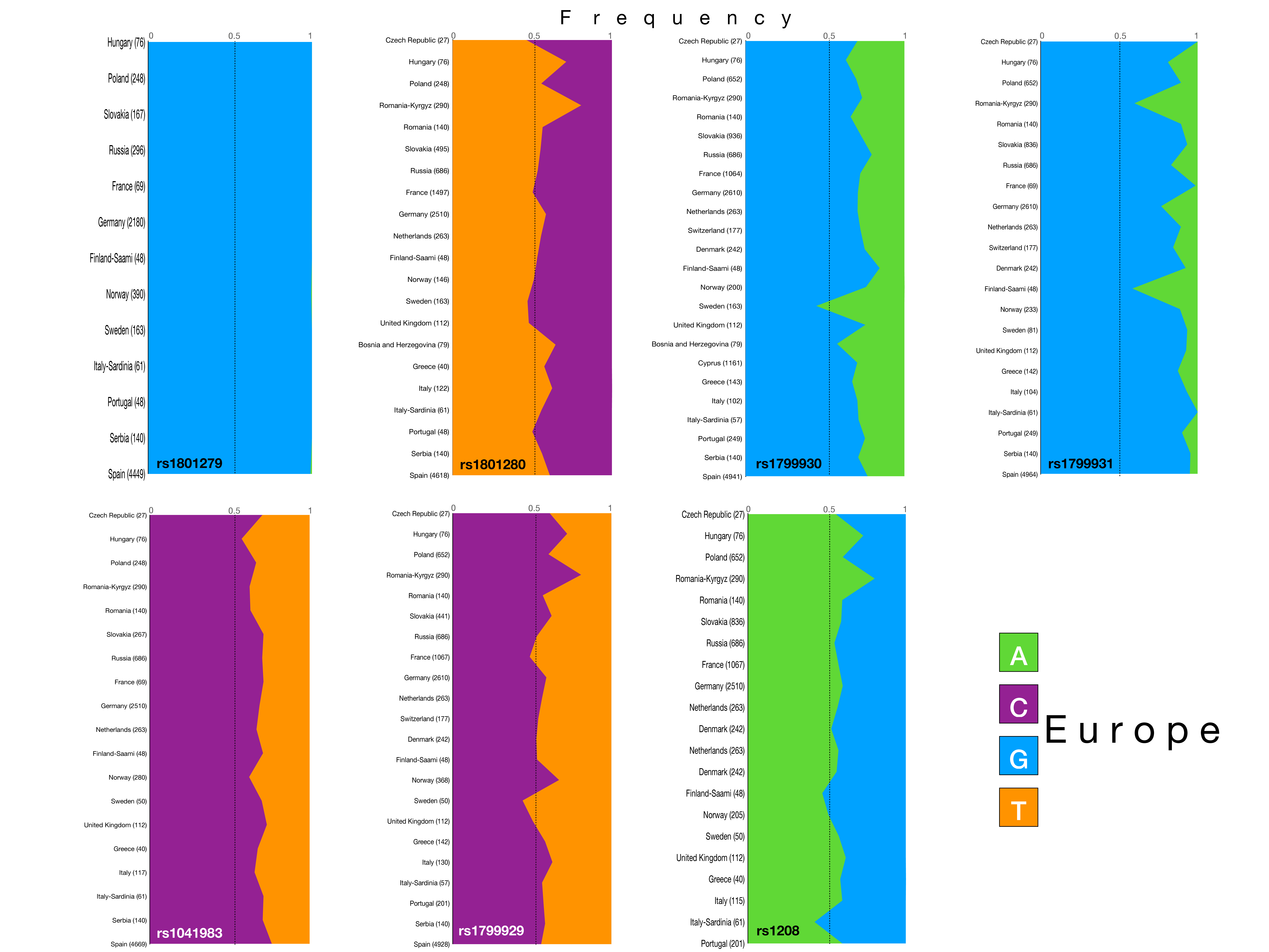

Supplement: S4 Fig — Note: A, Adenine; C, Cytosine, G, Guanine; T, Thymine. (TIFF) [file pone.0283726.s004.tiff]

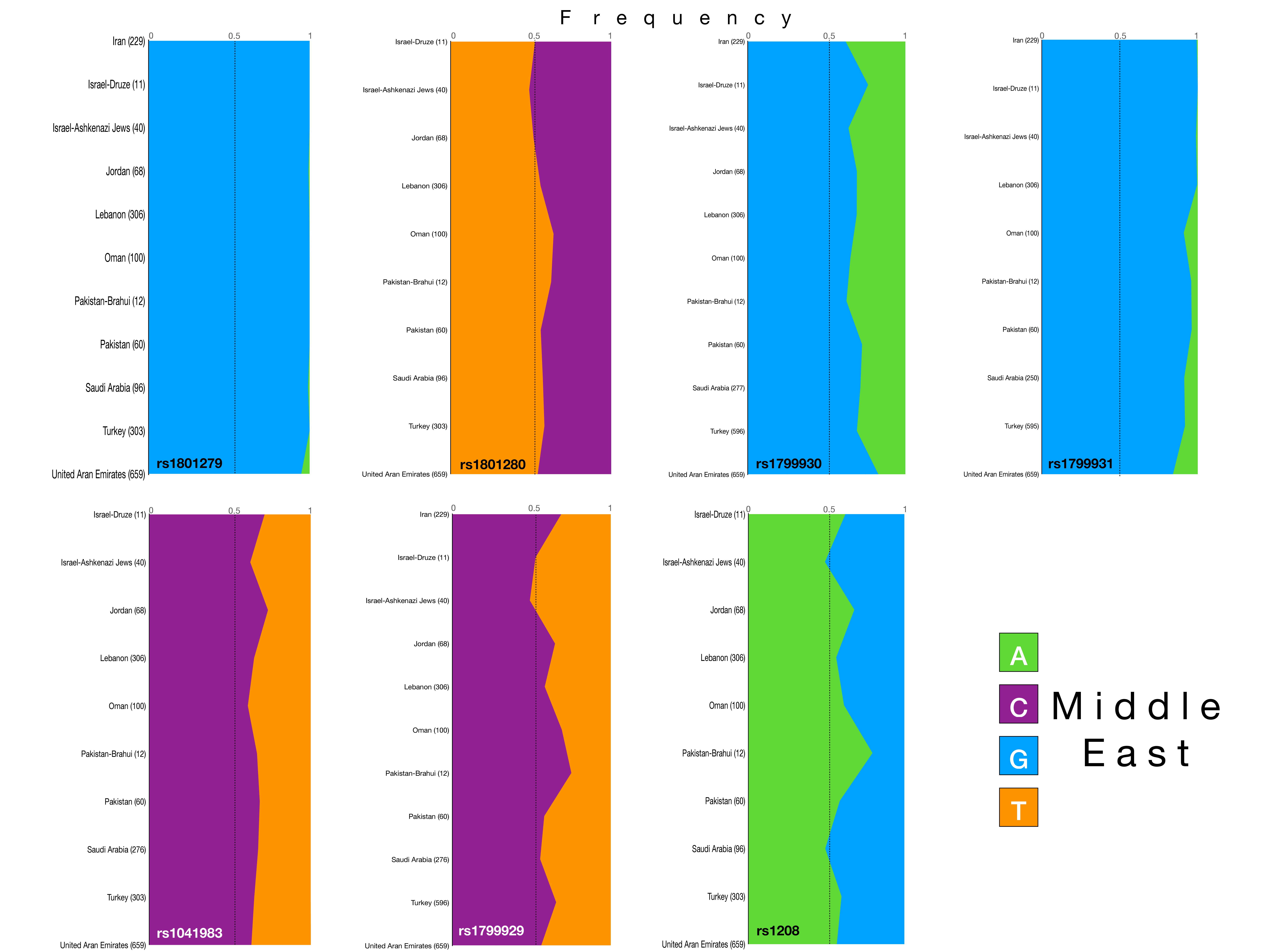

Supplement: S5 Fig — Note: A, Adenine; C, Cytosine, G, Guanine; T, Thymine. (TIFF) [file pone.0283726.s005.tiff]

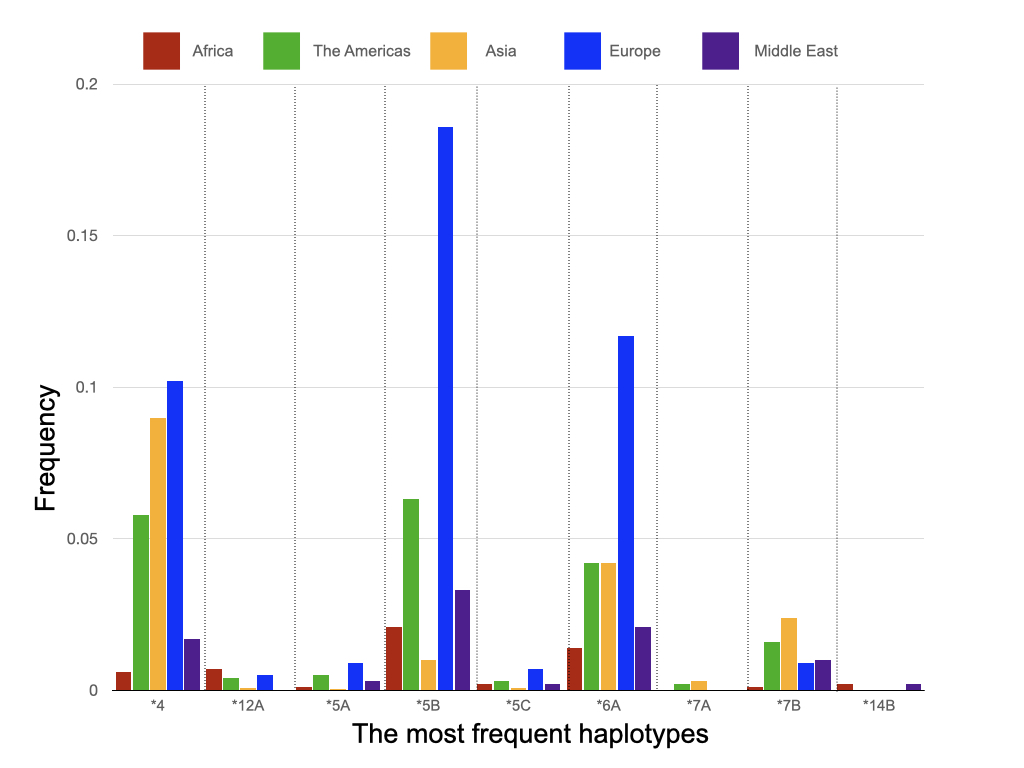

Supplement: S6 Fig — (TIF) [file pone.0283726.s006.tif]

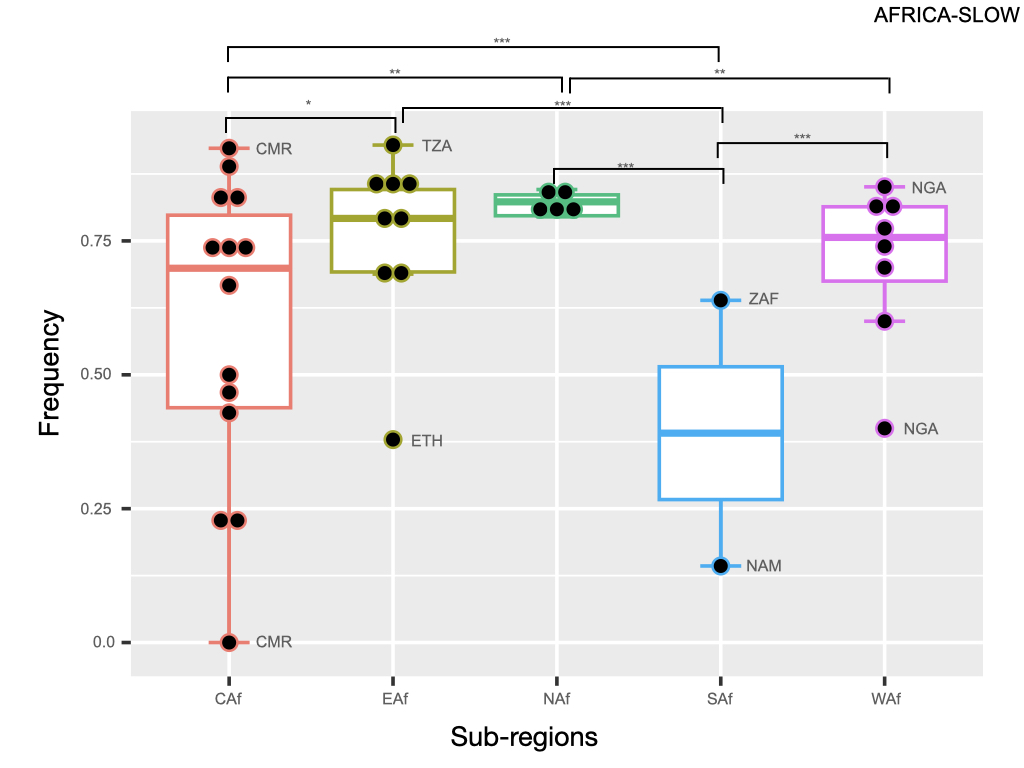

Supplement: S7 Fig — Note: CAf, Central Africa; EAf, East Africa; NAf, North Africa; SAf, South Africa; Waf, West Africa. CMR, Cameroon; ETH, Ethiopia; NAM, Namibia; NGA, Nigeria; TZA, Tanzania; ZAF, South Africa. (TIF) [file pone.0283726.s007.tif]

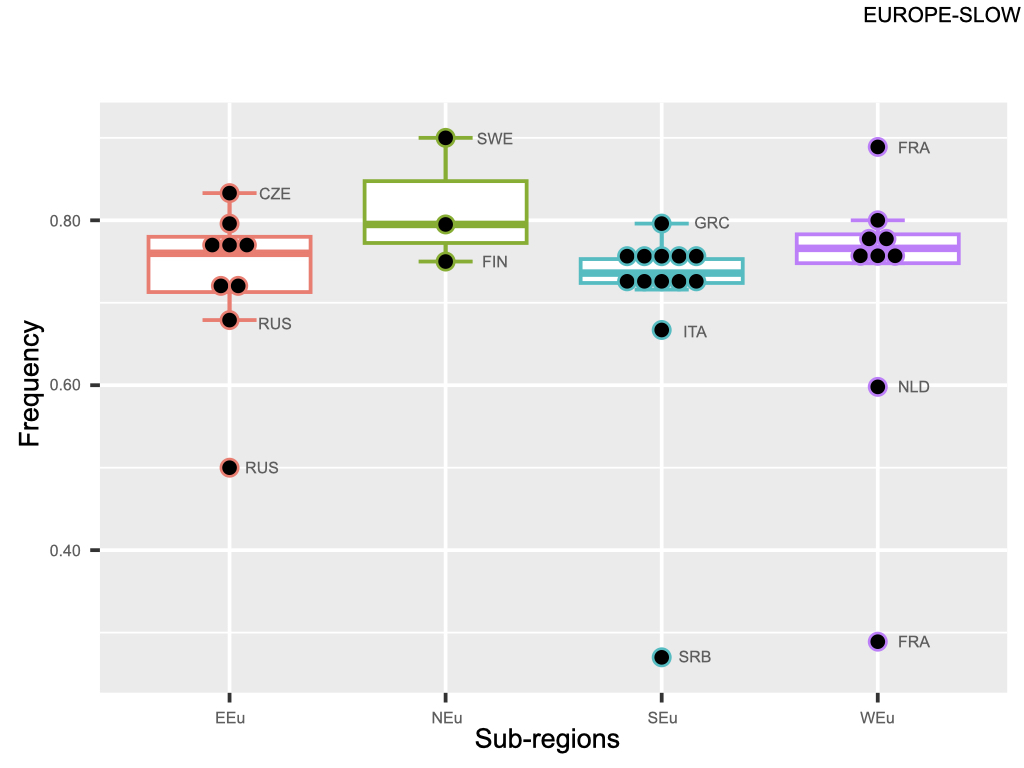

Supplement: S8 Fig — Note: EEu, East Europe; Neu, North Europe; SEu, South Europe; WEu, West Europe. CZE, Czech Republic; FIN, Finland; FRA, France; GRC, Greece; ITA, Italy; NLD, the Netherlands; RUS, the Russian Federation; SRB, Serbia; SWE, Sweden. (TIF) [file pone.0283726.s008.tif]

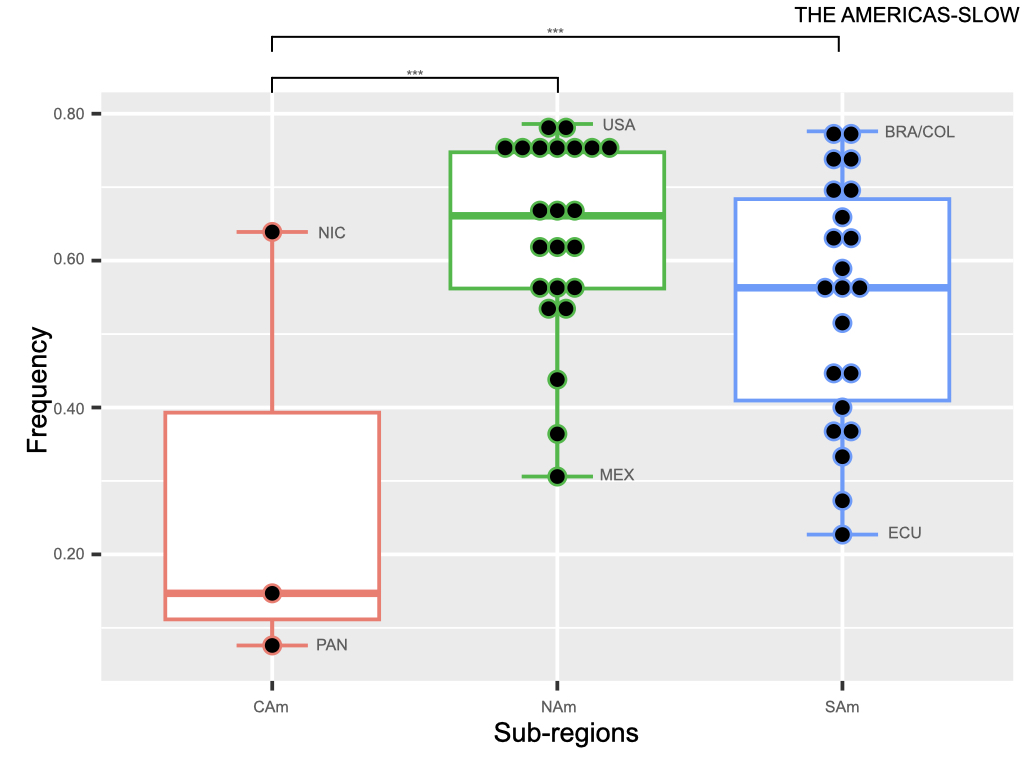

Supplement: S9 Fig — Note: CAf, Central America; Nam, North America; Sam, South America. BRA, Brazil; COL, Colombia; ECU, Ecuador; MEX, Mexico; NIC, Nicaragua; PAN, Panama; USA, the United States of America. (TIF) [file pone.0283726.s009.tif]

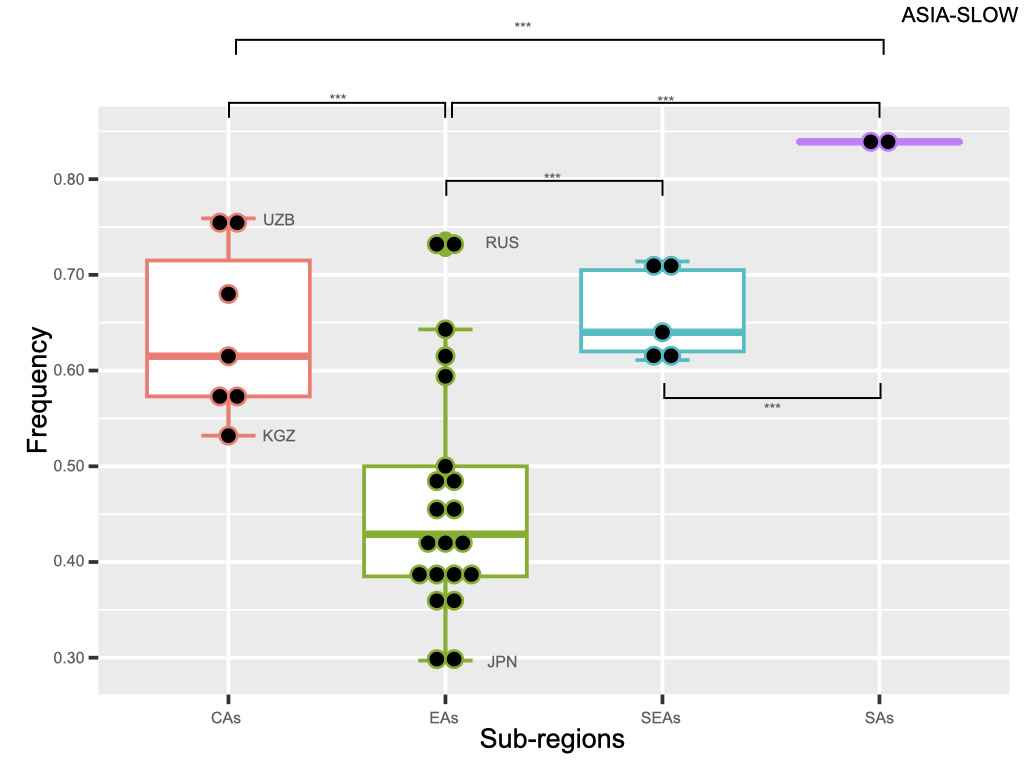

Supplement: S10 Fig — Note: Cas. Central Asia; EAs, East Asia; SEAs, Southeast Asia; SAs, South Asia. JPN, Japan; KGZ, Kirghizstan; RUS, the Russian Federation; UZB, Uzbekistan. (TIF) [file pone.0283726.s010.tif]

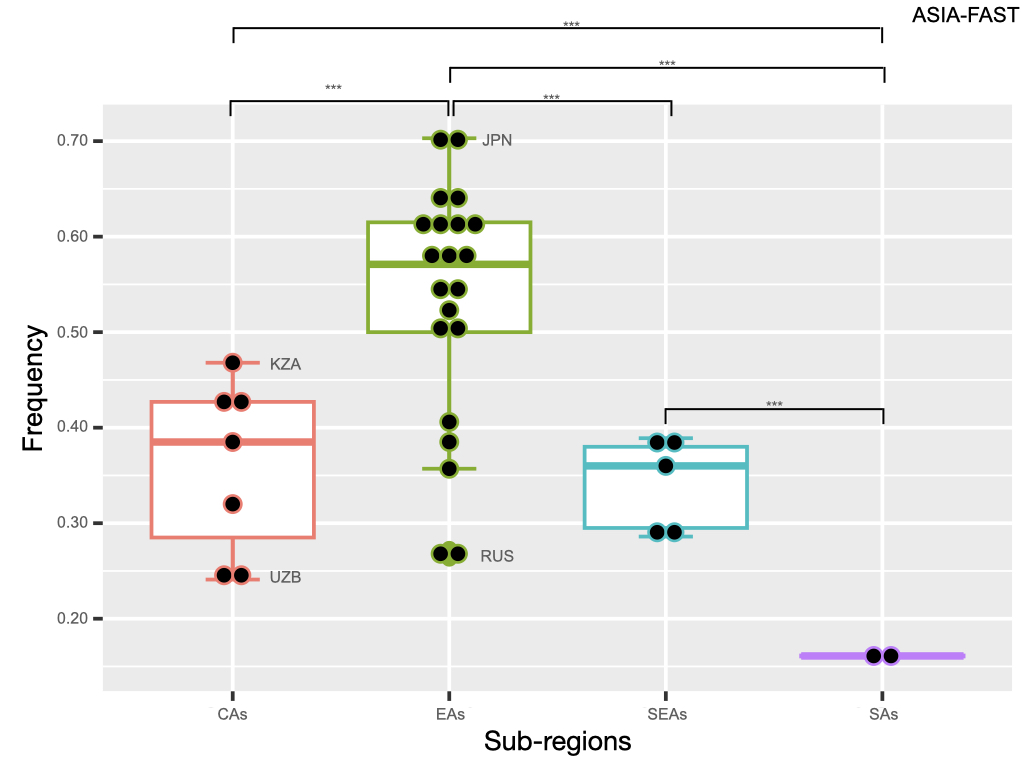

Supplement: S11 Fig — Note: Cas. Central Asia; EAs, East Asia; SEAs, Southeast Asia; SAs, South Asia. JPN, Japan; KGZ, Kirghizstan; RUS, the Russian Federation; UZB, Uzbekistan. (TIF) [file pone.0283726.s011.tif]

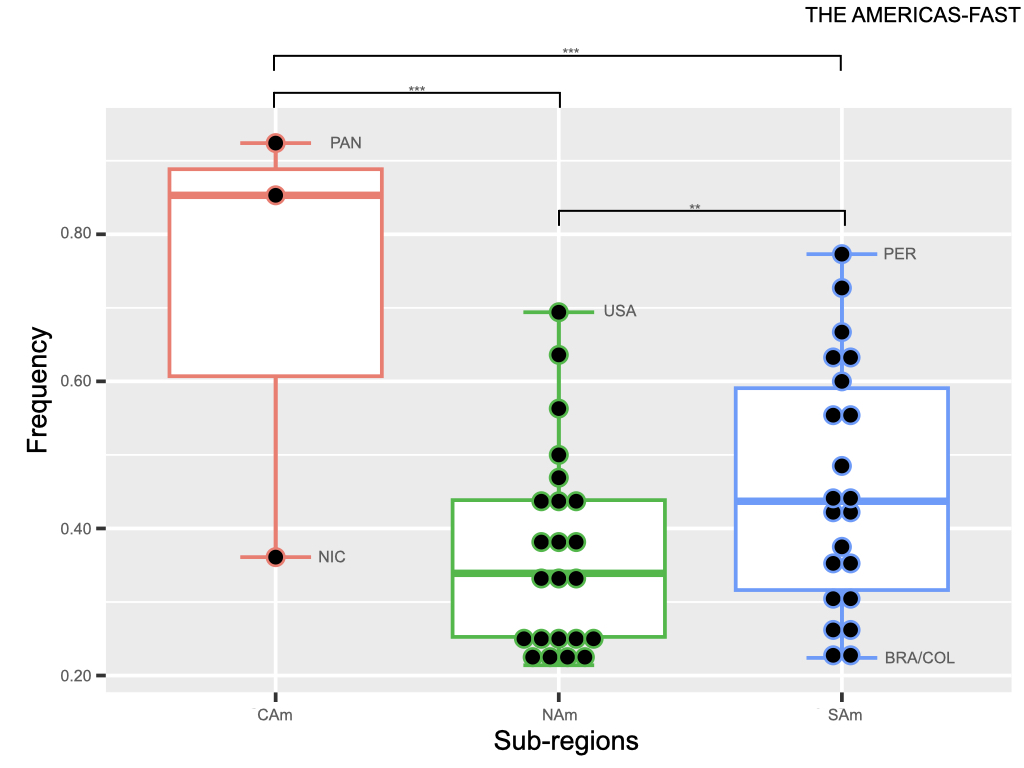

Supplement: S12 Fig — Note: CAf, Central America; Nam, North America; Sam, South America. BRA, Brazil; COL, Colombia; ECU, Ecuador; NIC, Nicaragua; PAN, Panama; USA, the United States of America. (TIF) [file pone.0283726.s012.tif]

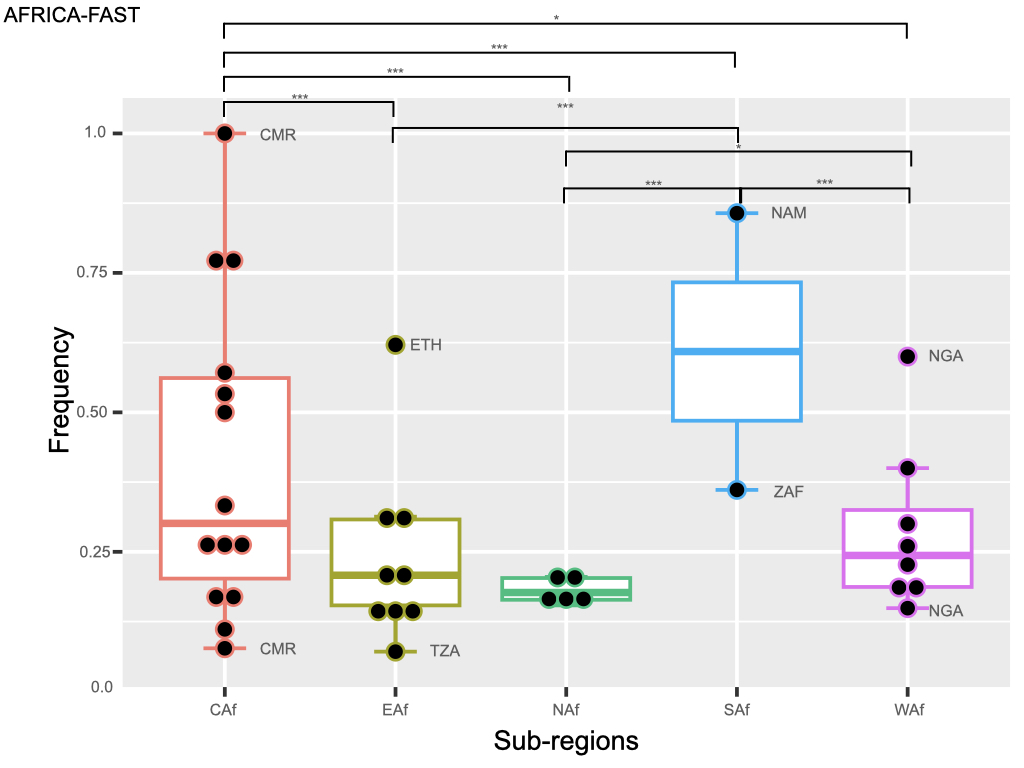

Supplement: S13 Fig — Note: CAf, Central Africa; EAf, East Africa; NAf, North Africa; SAf, South Africa; Waf, West Africa. CMR, Cameroon; ETH, Ethiopia; NAM, Namibia; NGA, Nigeria; TZA, Tanzania; ZAF, South Africa. (TIF) [file pone.0283726.s013.tif]

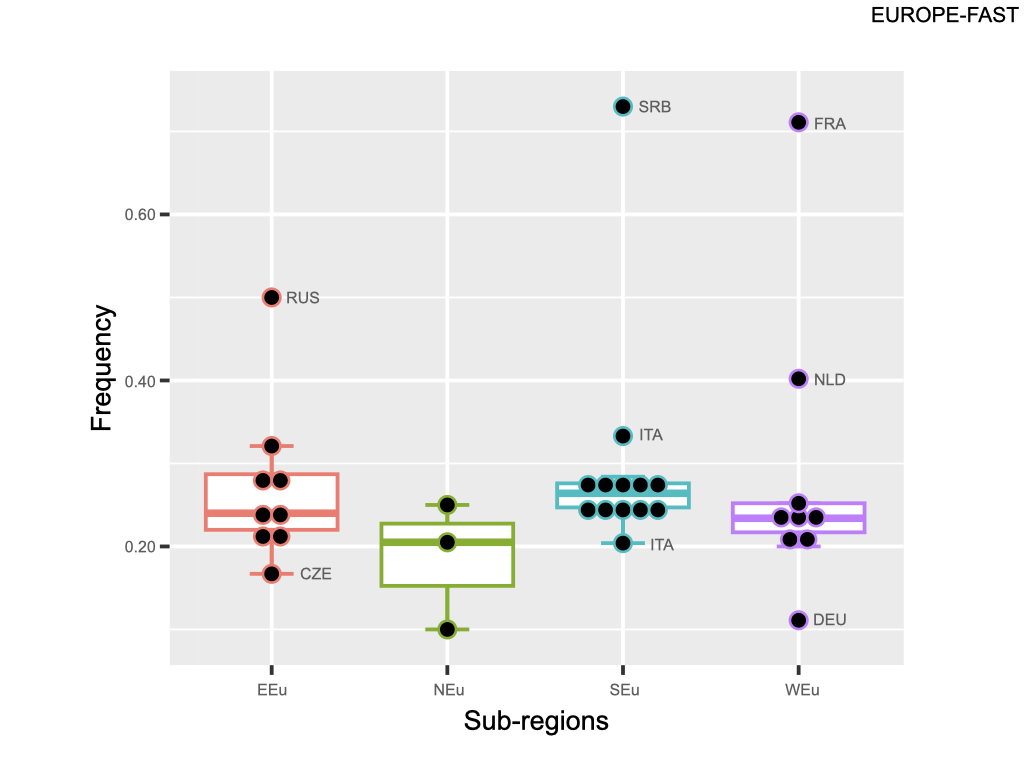

Supplement: S14 Fig — Note: EEu, East Europe; Neu, North Europe; SEu, South Europe; WEu, West Europe. CZE, Czech Republic; DEU, Germany; FRA, France; ITA, Italy; NLD, the Netherlands; RUS, the Russian Federation; SRB, Serbia. (TIF) [file pone.0283726.s014.tif]
